# Absence of Gut Microbiota Is Associated with RPE/Choroid Transcriptomic Changes Related to Age-Related Macular Degeneration Pathobiology and Decreased Choroidal Neovascularization

**DOI:** 10.3390/ijms23179676

**Published:** 2022-08-26

**Authors:** Jason Y. Zhang, Bingqing Xie, Hugo Barba, Urooba Nadeem, Asadolah Movahedan, Nini Deng, Melanie Spedale, Mark D’Souza, Wendy Luo, Vanessa Leone, Eugene B. Chang, Betty Theriault, Dinanath Sulakhe, Dimitra Skondra

**Affiliations:** 1 Department of Ophthalmology and Visual Science, University of Chicago, Chicago, IL 60637, USA; 2 Department of Medicine, University of Chicago, Chicago, IL 60637, USA; 3 Department of Pathology, University of Chicago, Chicago, IL 60637, USA; 4 Department of Ophthalmology and Visual Science, Yale University School of Medicine, New Haven, CT 06510, USA; 5 Animal Resources Center, University of Chicago, Chicago, IL 60637, USA; 6 Duchossois Family Institute, University of Chicago, Chicago, IL 60637, USA; 7 Department of Animal Biologics and Metabolism, University of Wisconsin, Madison, WI 53706, USA; 8 The Microbiome Center, University of Chicago, Chicago, IL 60637, USA; 9 Department of Surgery, University of Chicago, Chicago, IL 60637, USA

**Keywords:** age-related macular degeneration, gut microbiome, germ-free mice, RPE–choroid, RNA sequencing, choroidal neovascularization, angiogenesis, microglia, gut–retina axis

## Abstract

Studies have begun to reveal significant connections between the gut microbiome and various retinal diseases, including age-related macular degeneration (AMD). As critical supporting tissues of the retina, the retinal pigment epithelium (RPE) and underlying choroid play a critical role in retinal homeostasis and degeneration. However, the relationship between the microbiome and RPE/choroid remains poorly understood, particularly in animal models of AMD. In order to better elucidate this role, we performed high-throughput RNA sequencing of RPE/choroid tissue in germ-free (GF) and specific pathogen-free (SPF) mice. Furthermore, utilizing a specialized laser-induced choroidal neovascularization (CNV) model that we developed, we compared CNV size and inflammatory response between GF and SPF mice. After correction of raw data, 660 differentially expressed genes (DEGs) were identified, including those involved in angiogenesis regulation, scavenger and cytokine receptor activity, and inflammatory response—all of which have been implicated in AMD pathogenesis. Among lasered mice, the GF group showed significantly decreased CNV lesion size and microglial infiltration around CNV compared to the SPF group. Together, these findings provide evidence for a potential gut–RPE/choroidal axis as well as a correlation with neovascular features of AMD.

## 1. Introduction

Vision loss is among the top ten disabilities in adults, and age-related macular degeneration (AMD) is the most common cause of irreversible blindness in the industrialized world [1]. Beyond the modest effects of AREDS formula [2] and anti-*VEGF* agents [3], there are currently no preventative or therapeutic measures to effectively slow or reverse the progression of AMD.

Environmental factors, including diet and lifestyle, have been widely established as significant players in AMD pathobiology [4]. In patient cohorts, consumption of high-fat diets is positively associated with AMD risk and progression [5,6], and increased body mass index has a dose-dependent relationship with AMD risk [7]. The relationship between increased waist/hip ratio and incidence of AMD further reaffirms obesity as a risk factor [8]. However, despite these results, the mechanistic pathways behind the associations have yet to be fully revealed.

Considering this, studies have begun to explore connections between the gut microbiome and AMD. The human gut ecosystem is significantly shaped by diet and includes a vast network of microorganisms that interact to maintain human homeostasis [9]. Crosstalk both between microbiota and with distal organs has been well-established, linking gut dysbiosis with neurological, respiratory, and cardiovascular diseases [10]. This connection extends to the eye. Recently, it has been demonstrated that high-fat diets exacerbate AMD-associated choroidal neovascularization by altering gut microbiota composition and elevating local inflammation [11]. This is supported by additional data showing that a high glycemic diet increased AMD-associated features and changed plasma metabolite composition [12]. In the clinical context, patients with recent onset neovascular AMD had compositional and functional variations in their gut microbiome compared to matched controls [13]. Coupled with the unique transcriptomic profile recently identified by our group in germ-free mouse retinas [14], these results collectively suggest the presence of a gut–retina axis and a significant role in AMD pathogenesis.

As critical supporting tissues of the retina, the retinal pigment epithelium (RPE) and underlying choroid maintain photoreceptor health, and their degeneration play a significant role in both dry and wet forms of AMD [15]. While the precise molecular mechanism of this degeneration is not fully understood, the collection of microarray [16] and single-cell RNA-seq [17] data in RPE/choroid tissue is growing [18]. However, the current literature focuses on retinal transcriptomic changes in AMD patients, and there is little known about connections between the microbiome and RPE/choroid—particularly in animal models of AMD. Furthermore, studies utilizing germ-free (GF) animal models in this area are lacking. Considered the gold standard, GF animal models have been foundational in understanding the relationship between the microbial organ and states of homeostasis and disease in various tissues.

**Figure 1:**
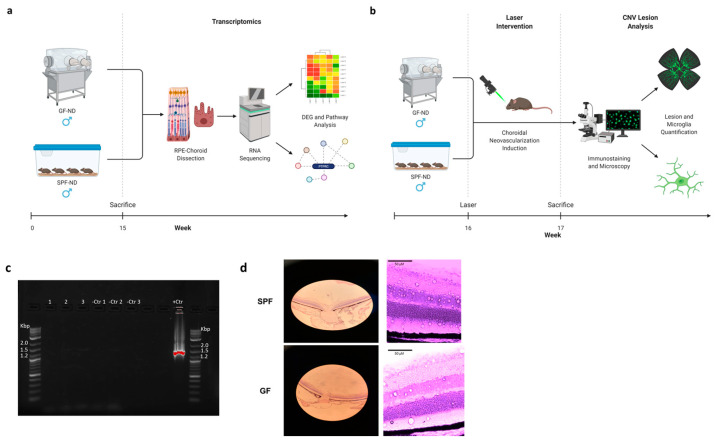
(**a**,**b**) Schematic diagrams indicating methodology for transcriptomic (**a**) and CNV lesion analysis (**b**) of GF-ND and SPF-ND mice. Images designed with Biorender. (**c**) Sterility of GF-ND mice (1–3) was confirmed by gel electrophoresis of fecal DNA RT-PCR products, with PCR mix and water (Ctr1), water (Ctr2), known negative sample (Ctr3), and known positive sample (+Ctr) as controls. (**d**) SPF-ND and GF-ND mice had similar retinal morphology at baseline as indicated by hematoxylin and eosin (H&E) staining. Scale bar = 50 μm.

In this current study, we analyzed the transcriptome of the RPE/choroid in germ-free (GF) mice on normal diet (ND) compared to isogenic control specific pathogen-free (SPF) mice. Performing high-throughput RNA sequencing of the RPE/choroid in these two groups, we investigated the overall interaction between the microbial organ and RPE/choroidal tissue (Figure 1a). Furthermore, we studied the effect of the microbiome on neovascular AMD, utilizing a specialized laser-induced choroidal neovascularization (CNV) model we have developed for GF mice in order to examine differences in CNV size and inflammatory response (Figure 1b). Validity of this animal model was further demonstrated through several assays, including a sterility check of GF mice (Figure 1c) and characterization of retinal morphology to confirm there were no differences between SPF and GF mice at baseline (Figure 1d). Together, these results illustrate which RPE/choroidal genes and pathways are modulated by the microbiome and how they correlate with neovascular features of AMD, providing evidence for a potential gut–RPE/choroidal axis in addition to the gut–retina axis and its potential role in AMD.

## 2. Results

### 2.1. Absence of the Microbiome Changed the RPE/Choroid Transcriptome

**Figure 2:**
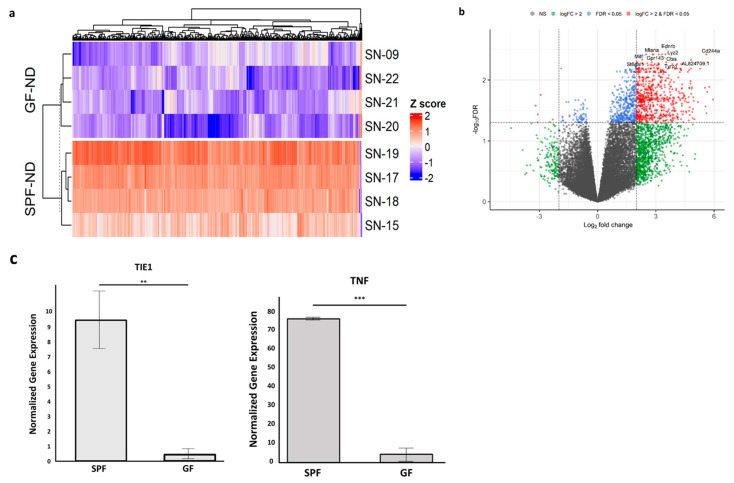
(**a**) Hierarchical clustering of RPE/choroidal genes between GF and SPF mice on normal diet. A total of 660 DEGs were identified with FDR < 0.05 and log_2_FC > 2. Red and blue indicate upregulated and downregulated genes, respectively. (**b**) Volcano plot detailing DEGs in the RPE/choroid of GF and SPF mice. A large majority of significant DEGs were downregulated in GF mice at FDR < 0.05 and log_2_FC > 2. (**c**) RT-qPCR of total RNA extracted from choroids of SPF (*n* = 3) and GF (*n* = 3) mice, validating identified DEGs (*TIE1*, *TNF*). Gene expression levels were normalized with GAPDH. ** *p* < 0.01, *** *p* < 0.001.

In order to examine the impact of the microbial organ on gene expression in the RPE/choroid, RNA sequencing was performed on GF-ND and SPF-ND mice, with four RPE/choroid samples per group. Using stringent criteria of FDR < 0.05 and log_2_FC > 2, we identified 660 highly significant differentially expressed genes (DEGs). A heatmap was plotted to show the hierarchical clustering of the DEGs (Figure 2a). A large majority of these genes were downregulated in the GF-ND mice compared to the SPF-ND group, with only six genes upregulated in the GF-ND group: *4933422A05RIK*, *MID1-PS1*, *DLL3*, *B230112G18RIK*, *ZFP988*, and *GCK* (Figure 2b). The top five downregulated genes in the GF-ND group, by log_2_FC value, were *CLEC3A*, *SELE*, *LILRB4A*, *ALKAL2*, and *CCL19*. At even stricter criteria of FDR < 0.01 and log_2_FC > 2, 130 significant DEGs were identified. At this cutoff, the top five downregulated genes in the GF-ND group were *SELE*, *CCL19*, *CD244A*, *FOXL1*, and *PATL2*. No genes were upregulated in the GF-ND group at this cutoff. A number of identified DEGs were validated by Real-Time Reverse Transcription-Polymerase Chain Reaction (RT-qPCR), including *TIE1* (log_2_FC = 4.42) and *TNF* (log_2_FC = 4.05), confirming a concordant decrease in expression in the GF-ND group (Figure 2c). A full list of genes and statistics are available in Supplementary Table S1.

### 2.2. Enrichment Analysis Revealed Key Biological Pathways Affected by the Microbiome including Angiogenesis and Immunological Activity

**Figure 3:**
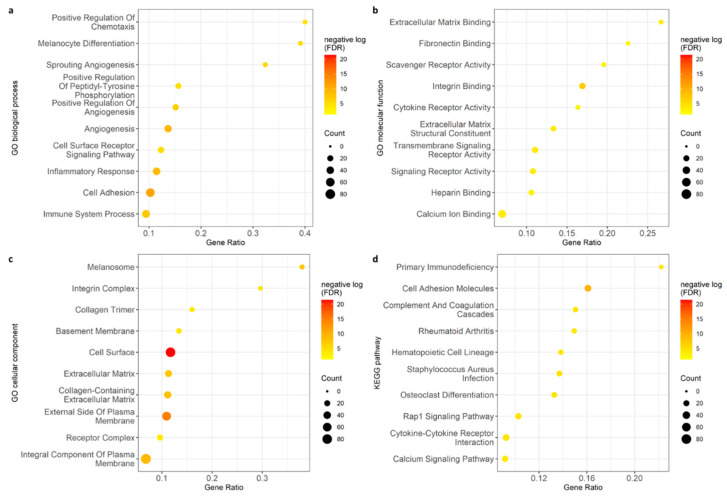
Gene ontology analysis with top 10 results for biological process (**a**), molecular function (**b**), cellular component (**c**), and KEGG pathway (**d**). Enrichment analysis showed multiple biological pathways affected, including angiogenesis and immunological activity.

Enrichment analysis used to identify Gene Ontology showed multiple biological pathways and functions downregulated in the GF-ND group. The most significant biological processes involved various angiogenesis mechanisms including sprouting angiogenesis alongside positive and negative regulation pathways. Also included among the top 10 affected biological processes were immune system processes and inflammatory response (Figure 3a). Other highly significant processes included melanocyte differentiation, chemotaxis regulation, and cell surface receptor signaling.

Highly affected molecular functions included fibronectin binding, scavenger receptor activity, and cytokine receptor activity (Figure 3b). Among cellular components affected, the most significant included melanosomes, integrin complexes, and receptor complexes (Figure 3c). The various vascular, immunological, and inflammatory components downregulated in the GF-ND group were further supported by the KEGG (Kyoto Encyclopedia of Genes and Genomes) pathway analysis. Within enriched KEGG pathways, significant changes were identified in complement and coagulation cascades, cytokine–cytokine receptor interactions, and primary immunodeficiencies (Figure 3d). A full list of enrichment data can be found in Supplementary Table S2.

### 2.3. Protein–Protein Associations Highlighted a Number of Hub Genes

**Figure 4:**
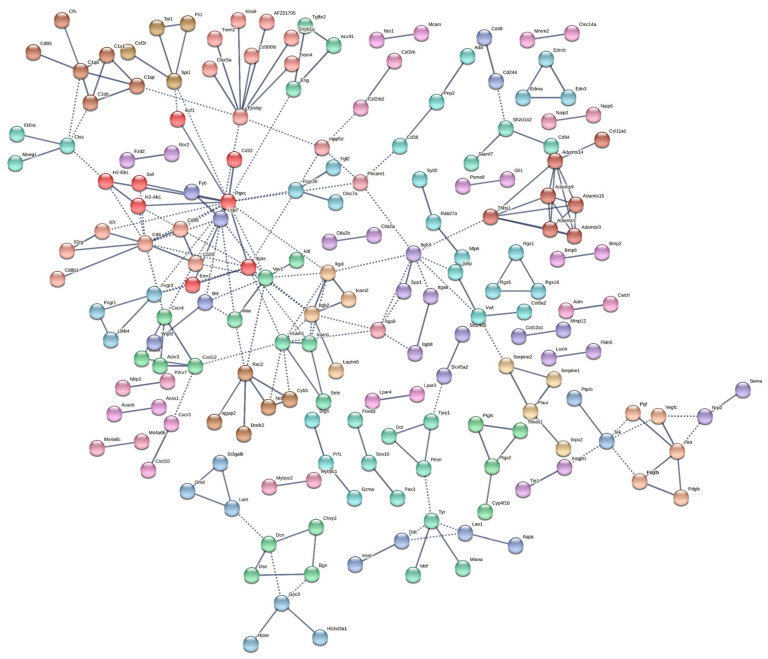
STRING network of protein–protein interaction (PPI) generated using identified DEGs (FDR < 0.05, log_2_FC > 2). Network was generated using STRING version 11.5.

Using the STRING database, we explored the protein-level functional connections within the 660 highly significant DEGs. At the strictest confidence level of 0.9, a protein network of 608 DEGs was mapped with an average node degree of 0.773 and average local clustering coefficient of 0.212 (Figure 4). The resulting PPI network was significantly enriched with *p*-value less than 1.0 × 10^−16^ and contained 235 edges compared to an expected 48 edges. Predominant nodes (hub genes) were determined through a number of algorithms, including degree of connection, maximum neighborhood component (MNC), and maximal clique centrality (MCC). The top three hub genes by degree were *PTPRC* (19 connections), *CD4* (12 connections), and *TYROBP* (10 connections). Furthermore, *PTPRC*, *CD4*, *ITGAX*, and *VCAM1* were among the top 10 hub genes across all three ranking methodologies (Table 1).

### 2.4. Germ-Free Mice Had Decreased CNV Lesion Size and Peripheral Microglia Activation

**Figure 5:**
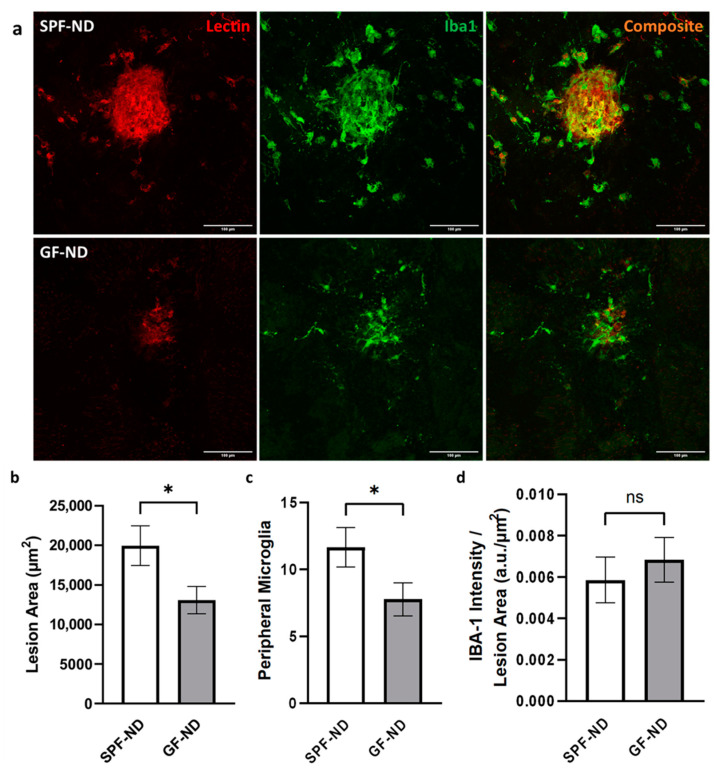
(**a**) Representative confocal images of laser-induced CNV lesions in SPF-ND and GF-ND mice. Scale bar = 100 μm. (**b**–**d**) Quantification of CNV lesion area (**b**), microglia surrounding the lesion (**c**), and IBA-1 signal intensity within given lesion area (**d**). GF-ND mice showed a reduction in both CNV lesion area and peripheral microglia activation (*p* < 0.05). * *p* < 0.05, ns: not statistically significant.

In the laser-induced CNV mouse model, the GF group showed a significantly smaller lesion area compared to their SPF-ND counterpart (Figure 5b). The average lesion area (±SEM) in the SPF-ND group was 19,955.35 ± 2512.21 μm^2^, and it was 13,093.37 ± 1735.68 μm^2^ in the GF-ND group (*p* = 0.029; *n* = 20, *n* = 9, respectively). Around the lesion, GF mice showed significantly lower peripheral microglia activation as well (Figure 5c). The SPF-ND group had 11.66 ± 1.48 IBA-1+ microglia compared to 7.78 ± 1.24 IBA-1+ microglia in the GF-ND group (*p* = 0.047). IBA-1 signal intensity (arbitrary unit, a.u.) within a given CNV lesion area showed no significant difference (*p* = 0.53; Figure 5d) between the SPF-ND group (0.0059 ± 0.0011 μm^−2^) and GF-ND group (0.0068 ± 0.0011 a.u./μm^2^).

## 3. Discussion

Compared to other complex treatment methods, the relative accessibility of the gut microbiota permits exciting opportunities for intervention in a number of vision-threatening diseases. Previous literature has revealed the impact of high-fat diet and other environmental factors on the progression of AMD [4,5,6,7], and more recently, the involvement of the gut microbiota in driving AMD pathogenesis [11,12,13,14]. However, the mechanism of this involvement, particularly at the transcriptomic level, has been limited and still largely uncharacterized. Furthermore, the use of germ-free (GF) animal models, the gold standard in microbiome studies, has been lacking. As a result, this study is one of the first, to our knowledge, in utilizing a GF animal model to demonstrate the possible existence of a distinct gut microbiome–RPE/choroid axis, as well as its associated transcriptomic components and effects on neovascular AMD formation. Using high-throughput RNA sequencing to profile the chorioretinal transcriptome of GF and SPF mice, we identified 660 DEGs, highlighting a number of biological pathways of interest. Subsequently, we showed that the absence of the microbiome was associated with decreased neovascular lesion formation and associated inflammation.

Among the identified transcriptomic differences, enriched pathways included the regulation of angiogenesis, vasculogenesis, and blood vessel morphogenesis. Aberrant angiogenesis has long been known to be closely tied to neovascular AMD, with budding choriocapillaris invading Bruch’s membrane and eventually destroying RPE and photoreceptors [19]. As a result, many common AMD treatments today involve the use of various anti-VEGF agents to slow or decrease the vision loss associated with CNV [20,21,22]. However, this therapy is temporary, and the consequences of its long-term use have been debated [23,24]. Furthermore, most importantly, no interventions exist to prevent CNV formation besides the limited effect of multivitamin supplements in a subgroup of patients [25]. In addition to the well-known *VEGFC*, our results from this study indicate a number of other DEGs involved in the regulation of angiogenesis that may serve as therapeutic targets. One significantly downregulated gene in GF mice was *CYP1B1*, a member of the cytochrome P450 family. *CYP1B1* is constitutively expressed in retinal vascular cells and is involved in ischemia-mediated retinal neovascularization [26]. This has previously been shown in *CYP1B1*^−/−^ mice, which displayed an attenuation of pathologic retinal angiogenesis [27]. Furthermore, the DEG *CXCR3* is connected to the inhibition of angiogenesis through *IP-10* [28], and a dysregulation of the chemokine receptor has been seen in patients with neovascular AMD [29]. Finally, two other DEGs, *TIE* and *ANGPT* (angiopoietin), are known to be tightly involved together in the regulation of vascular homeostasis and morphogenesis [30]. As a result, *TIE2* activation has been targeted to suppress CNV and relieve retinal hypoxic stress [31]. Coupled with the observed decrease in CNV formation in our GF mice, this relationship between the microbiome–RPE/choroidal axis and angiogenesis pathways appears to become stronger and clearer.

In addition, several genes involved in immune response and the complement cascade were found to have changed between the GF and SPF groups. The complement system plays an integral role in immune defense through inflammatory action and various lysis and clearance mechanisms. The past two decades have seen the identification of numerous AMD-associated polymorphisms in complement genes, beginning with complement factor H (*CFH*) [32,33]. Since then, the field has rapidly expanded and even led to recent clinical trials to address geographic atrophy in AMD patients given the C3 inhibitor pegcetacoplan [34]. Similarly, genes significantly affected in our study included *C3AR1* (Complement C3a Receptor 1), *CFH*, and *C1QA/B/C*. Other significantly affected immune response genes included *CD4* and *PTPRC* (CD45 antigen), which were also top-ranked hub genes in the protein–protein interaction analysis of DEGs (Table 1). Furthermore, a recent case–control study found the frequency of Th1 cells and *CXCR3*+ and *CD4*+ T-cells to be lower in patients with exudative AMD [35]. This further supports the view of AMD as a systemic disease—both through the microbiome and immune response.

The inflammatory response was also significantly affected between GF and SPF groups. Among the DEGs were *TNFRSF1B* (TNF Receptor Superfamily Member 1B) and *NLRP3* (NLR Family Pyrin Domain Containing 3). TNF-α is a proinflammatory cytokine that has been targeted with infliximab to treat AMD-associated CNV [36]. Meanwhile, the *NLRP3* inflammasome has been shown to have a protective role in AMD through regulation by *IL-18* [37]. These inflammatory mediators, alongside previously described changes in angiogenesis/*VEGF* pathways, likely contribute to the observed reduction in CNV lesion area and total peripheral microglia seen in the GF-ND group. Moreover, the gut microbiome likely plays a central role in this contribution. In line with our observations, Andriessen and colleagues showed that high-fat diet (HFD)-fed mice treated with antibiotics have reduced CNV lesion sizes and microglial activation compared to HFD mice without antibiotic treatment, highlighting the microbiome’s role in ocular inflammation and CNV progression [11]. Furthermore, the microbiome has been found to impact microglial maturation, function, and migration, with GF mice displaying impaired microglial morphology and response [38,39]. These findings, along with our observations, support the microbiome’s importance in mediating CNV progression and inflammation. Nevertheless, establishing causation in such studies remains challenging, and the interplay between microbiota, angiogenesis, and inflammatory cells such as macrophages and lymphocytes is complex—a relationship that may not be fully characterized by only CNV and microglial quantification in choroidal flatmounts [40].

One point of interest is the distinct and more prominent role of the microbiome in the RPE/choroid compared to our previously published results in the retina, which showed a more limited transcriptomic profile involving different pathways, including *MAPK*, obesity/metabolic syndrome, and glucocorticoid receptor binding [14]. A possible explanation for the differences in affected pathways may involve a cell-type-specific mechanism. Furthermore, ocular immune privilege may protect the retina more than the RPE/choroid from systemic perturbations.

Although this study highlights a potential gut–RPE/choroidal axis and its associations with AMD, it is important to acknowledge its limitations. While the germ-free mouse model is the gold standard for microbiome studies, the absence of a microbiome leaves the possibility for confounding variables, including changes in immune development, metabolism, and overall physiology. Furthermore, the nature of RNA sequencing limits studies of direct protein activity or cell-type-specific effects—but does provide invaluable guidance for these future confirmatory studies. Finally, the laser-induced CNV model does not fully typify neovascular AMD, as human retina anatomy and physiology are unique compared to animals. Nevertheless, these findings suggest that a connection between the microbiome and RPE/choroid may exist and potentially play a role in AMD. Future studies are needed to more precisely define this relationship, delineate its involvement in the pathobiology of AMD and other retinal diseases, and identify avenues for therapeutic intervention through gut microbiome alterations.

## 4. Materials and Methods

### 4.1. Animals

All animal procedures were performed per the Association for Research in Vision and Ophthalmology (ARVO) guidelines for animal use in ophthalmic and vision research and were approved by the Institutional Animal Care and Use Committee (IACUC) at the University of Chicago (UChicago). Isogenic adult male C57Bl/6J SPF and GF mice were utilized for this study. SPF mice were bred in the Animal Resources Center (ARC) at the UChicago animal vivarium. In order to ensure sterility, GF mice were housed, bred, and maintained in a sterile flexible film isolator at the Gnotobiotic Research Animal Facility (GRAF) at UChicago. Sterility was further ensured by autoclaving diets (121 °C, 30 min) for GF mice and monitoring biweekly fecal pellets via microbial cultures as explained by Theriault et al. [41]. Furthermore, as the confirmatory test, RT-PCR was performed on DNA of the fecal samples for 16S rRNA (Figure 1c). Total DNA extraction was performed using the DNeasy PowerSoil kit (Qiagen, Hilden, Germany). Primers 27F and Rp2 were used for PCR to obtain a 1500 base pair (bp) product from bacterial 16S ribosomal DNA.

Standard environmental parameters [42] were maintained with 12-h light cycles. Rodent chow and water were provided ad libitum to all mice. For RNA sequencing, four mice were used for each group (SPF and GF) and sacrificed at an average age of 15 weeks via CO_2_ asphyxiation followed by cervical dislocation; all measures were taken to minimize stress and pain throughout the procedure. For laser surgery, SPF (*n* = 20) and GF (*n* = 9) mice were sacrificed at an average of 17 weeks via CO_2_ asphyxiation followed by cervical dislocation.

### 4.2. Tissue Preparation and Hematoxylin and Eosin (H&E) Staining

Globes were dissected and cryopreserved using previously published methods [43]. The eyes were cryopreserved and serially sectioned with 8–10-micron thickness. H&E staining was performed on sagittal sections to assess histomorphologic changes using light microscopy as described before [44]. In brief, the slides were immersed in water for 30 s, then dipped into a Coplin jar containing Mayer’s hematoxylin (MilliporeSigma, Burlington, MA, USA) and agitated for 30 s and rinsed for one minute. Slides were subsequently placed in 1% eosin Y solution (MilliporeSigma, Burlington, MA, USA) for 10–30 s with agitation and then dehydrated with two changes of 95% alcohol and two changes of 100% alcohol for 30 s each, and two changes of xylene; they were then mounted with one drop of mounting medium (Vector Labs, Newark, CA, USA) and covered with a coverslip. The slides were scanned using the Phillips IntelliSite whole-slide scanner.

### 4.3. RNA Extraction

Upon mouse sacrifice, eyes were immediately enucleated and dissected. RPE/choroid tissue was extracted under appropriate sterility and decontamination techniques with RNase ZAP (Thermo Fisher Scientific, Waltham, MA, USA) used on all surfaces and equipment. RNAlater (Qiagen) was used to store tissue at −80 °C until RNA extraction. Four samples of purified RNA were collected for each group using RNeasy (Qiagen). RNA concentration was confirmed for each sample before sequencing using NanoDrop 2000cc (Thermo Fisher Scientific, Waltham, MA, USA).

### 4.4. RNA Sequencing

Before sequencing, all samples were confirmed to have the required RNA integrity number (RIN), with quality control conducted via Bioanalyzer (completed at the UChicago Genomics Core). cDNA libraries for each sample were prepared using Tru-Seq RNA Sample Prep Kits (Illumina, San Diego, CA, USA) for 100 bp paired-end reads. Libraries were multiplexed and sequenced on NovaSEQ6000 (Illumina) using PE100bp, and data were imported in R for bioinformatics analysis.

### 4.5. Statistical Analysis

The raw fastq files generated from the RNA-seq analysis were examined by the fastqc to evaluate the quality. The STAR [45] (version 2.4.2a, Stanford University, Stanford, CA, USA) aligner was used to map the raw reads to the reference mouse genome (GRCm38). The STAR default parameter for the maximum mismatches was 10, which is optimized based on mammalian genomes and recent RNA-seq data. The genetic features from Gencode [46] vM23 were extracted from the resulting bam file produced by STAR. The raw gene expression count matrix was then generated by featureCounts [47] (version subread-1.4.6-p1).

We obtained a matrix with 55,228 genetic features and 8 samples as the final output. The data were imported to R for downstream analysis. The raw gene count matrix for all samples was transformed into log-CPM (count per million) values. Genes without sufficiently large counts were filtered by the filterByExpr function using EdgeR [48]. The low-expressed genes were defined as those with read counts <10 in samples within each group and <15 in all samples in order to filter the background noise. In total, 24,784 genes were retained for the downstream analysis. We used Limma [49,50] Voom normalization to standardize the gene expression matrix, where the raw library sizes were scaled using TMM (trimmed mean of M values). Multidimensional scale plots were generated on the top two leading FC dimensions that showed the similarity between samples.

The differential expression analysis was performed on contrast group SPF against GF using Limma [49]. Significant differentially expressed genes (DEGs) were extracted with FDR adjusted *p*-value < 0.05 and log2FC > 2, and 120 pseudogenes were removed from the list. In total, 660 genes were identified as DEGs for the further downstream analysis. The enrichment analysis through LYNX [51] API was used to find the over-represented gene ontology (GO) terms in molecular functions, biological processes, and cellular component subcategories, as well as the enriched KEGG pathways. We applied FDR correction on raw *p*-values, and the significant enrichment results were selected based on FDR-adjusted *p*-value < 0.05.

To explore the protein-level functional connection of the identified DEGs, we mapped them into the STRING (Search Tool for the Retrieval of Interacting Genes) database, which contains a wealth of validated and text-mined protein–protein interactions among the proteins [52]. A significantly enriched protein network was obtained from 608 mapped DEGs. We applied the Markov clustering with inflation parameter = 3 and a confidence level of 0.9 to group the proteins by the network connections. Hub gene analysis was conducted with Cytoscape [53].

### 4.6. Real-Time Reverse Transcription–Polymerase Chain Reaction (RT-qPCR)

To validate results obtained by RNA-seq analysis, we performed RT-qPCR on SPF (*n* = 3) and GF (*n* = 3) choroid RNA samples extracted as previously described. HyperScript Reverse Transcriptase (APExBIO, Houston, TX, USA) kits were used to create cDNA from each of the target genes, *TNF* (FW: 5′-CCCCGCTTACAGTTCCTCTT-3′, REV: 5′-AGCAAAACGGGTTAGGAGGG-3′) and *TIE1* (FW: 5′-GACGCACCTAGGACCAAACA-3′, REV: 5′-GGTTCACACAGGAGTCAGGG-3′). Using PrimeTime Gene Expression Master Mix (Integrated DNA Technologies, IDT) and SYBR green (ThermoFisher), the qPCR was performed on CFX384 Real-Time Systems (BioRad). GAPDH (FW: 5′-TGAATTGTGCACGCACCAAG-3′, REV: 5′-GGGAAGCAGCATTCAGGTCT-3′) was utilized as a housekeeping gene to normalize the results, and triplicates of each gene were performed. The data were analyzed through BioRad CFX Maestro with normalized expression levels compared between groups.

### 4.7. Laser

Four CNV lesions were induced per mouse using 532 nm green laser (Iridex, Oculight GLx, Mountain View, CA, USA) at 50 μm spot size, 120 mW power, and 100 ms duration. Mice were lasered at an average age of 16 weeks for both groups. The GF lasering was performed under sterile conditions inside of a laminar flow hood, and all mice were sacrificed by CO_2_ inhalation one week after the laser session, as described in detail in our paper [54]. After the procedure, GF mice were placed in hermetically sealed, ventilated cages and kept under sterile conditions. Sterility checks were performed similarly as described earlier, through culturing of fecal pellets and confirmation by RT-PCR of fecal sample DNA for 16S rRNA.

### 4.8. Immunostaining and Imaging of Choroidal Flatmounts

Eyes selected for immunohistochemistry were fixed in 4% paraformaldehyde overnight at 4 °C. The eyes were dissected the following day, separating the retina and cornea, and placed in a donkey serum solution (5% Donkey Serum, 2.5% BSA, 0.5% Triton 100X in 1X TBS) at 4 °C overnight. Dissected retinas were incubated overnight in IBA-1 antirabbit antibody (1:500) at 4 °C. The following day, the retinas were washed six times, 10 min each, in TBS-T (0.5% Tween 20). Following this, the retinas were incubated in antirabbit FITC (Abcam ab6717, 1:400) and isolectin (ThermoFisher I21411, 1:100) for two hours at room temperature in the dark. Retinas were then washed again six times in TBS-T and mounted on glass slides with ProLong Gold antifade reagent (ThermoFisher P36930). After sealing with transparent nail polish and curing for an hour, the choroidal flatmounts were visualized with the Leica SP5 microscope (Leica Microsystems, Deerfield, IL, USA).

Confocal CNV lesion images were taken at 20× magnification at 488 nm (FITC/IBA-1) and 561 nm with a 2 μm Z-step. Images were analyzed on FIJI [55]. Maximum-intensity Z-stack values were used to create 2D images. CNV lesion size was delineated and measured on the lectin channel. The lesion outline was used to delimit and measure the intensity of IBA-1, providing the average signal within the lesion and highlighting active microglia outside the lesion for quantification. Lesion size, IBA-1 signal within lesion, and microglia count (within 150 µm around the lesion) were independently quantified by two graders and averaged, with outliers removed. GraphPad Prism version 9.3.1 was used for statistical analysis (GraphPad Software, San Diego, CA, USA), with two-tailed Welch’s *t*-test used to determine significance.

## Figures and Tables

**Table 1:** Top 10 Hub Genes (Score) in STRING Network Analysis by Methodology.

Degree	Maximum Neighborhood Component (MNC)	Maximal Clique Centrality (MCC)
Ptprc (19)	Ptprc (14)	Adamts14 (121)
Cd4 (12)	Cd4 (11)	Thbs1 (121)
Tyrobp (10)	Vav1 (7)	Adamts1 (120)
Vav1 (9)	Itgb2 (7)	Adamts15 (120)
Itgax (9)	Itgax (6)	Adamts9 (120)
Vcam1 (9)	Vcam1 (6)	Adamts13 (120)
Itgb2 (8)	Itgal (6)	Ptprc (43)
Itgb3 (8)	Lcp2 (5)	Cd4 (31)
Lcp2 (7)	Cd86 (5)	Itgax (20)
Itgal (7)	Icam1 (5)	Vcam1 (19)

## Data Availability

Complete dataset of identified DEGs is available in Supplementary Table S1. Complete dataset of enrichment data is available in Supplementary Table S2.

## Supplementary Materials

The supporting information can be downloaded at: https://www.mdpi.com/article/10.3390/ijms23179676/s1.
